# A Dictionary of Terms Used in Medicine and the Collateral Sciences

**Published:** 1836-04

**Authors:** 


Art. IX.—*A Dictionary of Terms used in Medicine and the Collateral Sciences.* By Richard D. Hoblyn, M.A. Oxon.—*London,* 1835. 12mo. pp. 329.

Mr. Hoblyn's object in compiling this Dictionary has been to present to the student in a concise form an explanation of the terms which are *most* used in medicine. He has introduced many of the more recent terms, and has supplied, in an Appendix, a small selection of obsolete ones. His work forms a duodecimo volume of 350 pages, closely printed in a very clear legible type. Forsyth's London Medical and Surgical Dictionary, to which this approaches the nearest, (printed in 1826,) is swollen to twice the size, chiefly by the insertion of terms which are never met with in the ordinary books of modern medicine.

Mr. Hoblyn has considerably simplified his subject by his arrangement of words having the same prefixes and affixes. Thus, all words compounded with the same preposition, or generic term, are classed under the same heading, by which the repetition of the same word is avoided, and the derivations themselves are more easily remembered. To take an instance:

"GASTER (γαστὴρ), the stomach.

1. *Gastric.* Appertaining to the stomach: hence the term *gastric juice,* or the digestive fluid of the stomach.
2. *Gastritis.* Inflammation of the stomach.
3. *Gastro-cele* (κήλη, a tumour.) Hernia of the stomach.
4. *Gastro-cnemii* (κνημη, the leg.) Muscles which principally form the calf, or belly of the leg: they are distinguished as *externus* and *internus,* and are attached, respectively, to the outer and inner condyle of the femur. Their office is to extend the foot. [Their insertion should not have been omitted.]
5. *Gastr-odynia* (οὐδνη, pain,) or Gastralgia (αλγος, pain.) Pain in the stomach.

6. *Gastro-enteritis.* Inflammation of the gastro-intestinal mucous membrane.
7. *Gastro-epiploic* (ἐπίπλοον, the omentum). Belonging to the stomach and omentum, as applied to a branch of the hepatic artery, lymphatic glands of the abdomen, &c.
8. *Gastro-raphe* (ῥαφή, a suture.) A suture uniting a wound of the belly, or some of its contents.
9. *Gastro-splenic omenta.* A term applied to the laminæ of the peritoneum, which are comprised between the spleen and the stomach.
10. *Gastro-tomia* (τομή, section.) The operation of opening the abdomen, as in the Cæsarian section." (P. 80.)

The classification of prefixes is introduced into the body of the work; but, as it would be impossible to do so with the affixes, a collection of words so arranged is given separately. For instance:

"— CARDIA (καρδία, the heart.) A termination denoting the heart. Hence—
*A-cardiac* (a, privative.) A term applied to animals without a heart.
*Hydro-cardia* (ὕδωρ, water.) Hydropericardia: dropsy of the pericardium.
*Peri-cardium* (περὶ, around.) The membrane which surrounds the heart."

These selections will give a fair notion of the merits of the explanations. The anatomical parts are clear and simple, no detail being attempted.

Although the primary object is medicine, yet the nomenclatures of the sciences more immediately connected with medicine have been introduced; as well as classifications of poisons and their antidotes, and various tables of physiology, materia medica, &c. compiled or copied from good authorities. Under the word Climate, a useful condensation of Dr. Clark's observations is given. A considerable collection of the formulæ of quack medicines has been made; and, although the proportions of the ingredients is not given in all instances, nor the authorities for the information, yet the information it affords may be often advantageous. Under the head of Injection, (in the Supplementary List,) the author has given the composition of the various injections employed for anatomical purposes, with directions for preparing and using them. Under the word Thermometer is given, among other information, a table shewing the correspondence of the three thermometers, called Fahrenheit's, the Centigrade, and that of Reaumur, which is continually useful as a reference to the numerous readers of French works, in which the temperature is always expressed according to the centigrade thermometer.

On the whole, Mr. Hoblyn has accomplished the object he had in view; and we can safely recommend his work to the younger students of medicine especially, who will find it a portable and very useful addition to their libraries. We would suggest that, in a future edition, some of the tables (such as physiology) might be advantageously omitted, as well as sundry quotations from Shakspeare, &c.; and that, in their stead, more pertinent information should be introduced: such as the synonyms of muscles, which are often very perplexing to the beginner, and for which he has recourse to his dictionary. Cloquet's Anatomie Descriptive, vol. i., would furnish these particulars. In some instances, also, such as Tendo Achillis, &c., we think that Mr. Hoblyn has been inclined to give the student credit for too much information; and that a little more detail in the explanation of the derivations should have been entered into; for the object of the work is to explain terms to those who, for the most part, are deficient in that sort of knowledge, which, when possessed, renders the consultation of these limited dictionaries rarely necessary. The quantities in the Latin words might also have been marked, as well as in such words as Plethora, &c., and the derivations of many of the metals, as Nickel, Palladium, &c.

